# Prevalence and thrombotic risk of SGLT-2 inhibitor-associated erythrocytosis: a retrospective cohort study

**DOI:** 10.1186/s12933-025-02805-6

**Published:** 2025-07-10

**Authors:** Ji Yun Lee, Ju-Hyun Lee, Eun-Jung Jung, Woochan Park, Jeongmin Seo, Minsu Kang, Eun Hee Jung, Sang-A Kim, Koung Jin Suh, Ji-Won Kim, Se Hyun Kim, Jeong-Ok Lee, Jin Won Kim, Yu Jung Kim, Keun-Wook Lee, Jee Hyun Kim, Soo-Mee Bang

**Affiliations:** Department of Internal Medicine, Seoul National University College of Medicine, Seoul National University Bundang Hospital, Gumi-ro 173 Beon-gil, Bundang-gu, Seongnam-Si, 13620 Gyeonggi-do Korea

**Keywords:** SGLT-2 inhibitors, Diabetes mellites, Erythrocytosis, Thrombosis

## Abstract

**Background:**

Sodium-glucose cotransporter 2 (SGLT-2) inhibitors, widely used for type 2 diabetes and cardiorenal conditions, may induce erythrocytosis, potentially increasing cardiovascular risk. This study investigates its prevalence, risk factors, and thrombotic implications.

**Methods:**

In a single-center retrospective study, we analyzed 6787 patients prescribed SGLT-2 inhibitors (2014–2024). Erythrocytosis was defined as hemoglobin > 16.5 g/dL or hematocrit > 49% in men, > 16.0 g/dL or > 48% in women. We assessed prevalence, risk factors, and thrombotic events using logistic regression.

**Results:**

Erythrocytosis occurred in 1145 patients (16.9%) over a median follow-up of 530 days (IQR, 277–981), with a median hemoglobin rise of 1.0 g/dL (IQR, 0.4–1.8). Male sex (OR 3.24, 95% CI 2.47–4.26), BMI ≥ 25 kg/m^2^ (OR 1.97, 95% CI 1.63–2.39), and current smoking (OR 2.41, 95% CI 1.96–2.96) significantly increased risk (all p < 0.001), while age ≥ 70 years, hypertension, dyslipidemia, and chronic kidney disease were associated with reduced risk. Thrombosis was rare (0.5%, 33 patients) and associated with antiplatelet use (OR 3.57, 95% CI 1.60–7.97), anticoagulant use (OR 5.93, 95% CI 2.60–13.57), and baseline erythrocytosis (OR 3.75, 95% CI 1.41–9.96). Among 33 patients with thrombosis, five exhibited erythrocytosis at the time of the event and within the prior six months; all had arterial thrombosis associated with underlying conditions (atrial fibrillation, coronary calcification, atherosclerosis), not directly attributable to SGLT-2-induced erythrocytosis.

**Conclusions:**

SGLT-2 inhibitors are associated with a 16.9% prevalence of erythrocytosis, but thrombotic risk appears primarily driven by pre-existing conditions.

**Graphical abstract:**

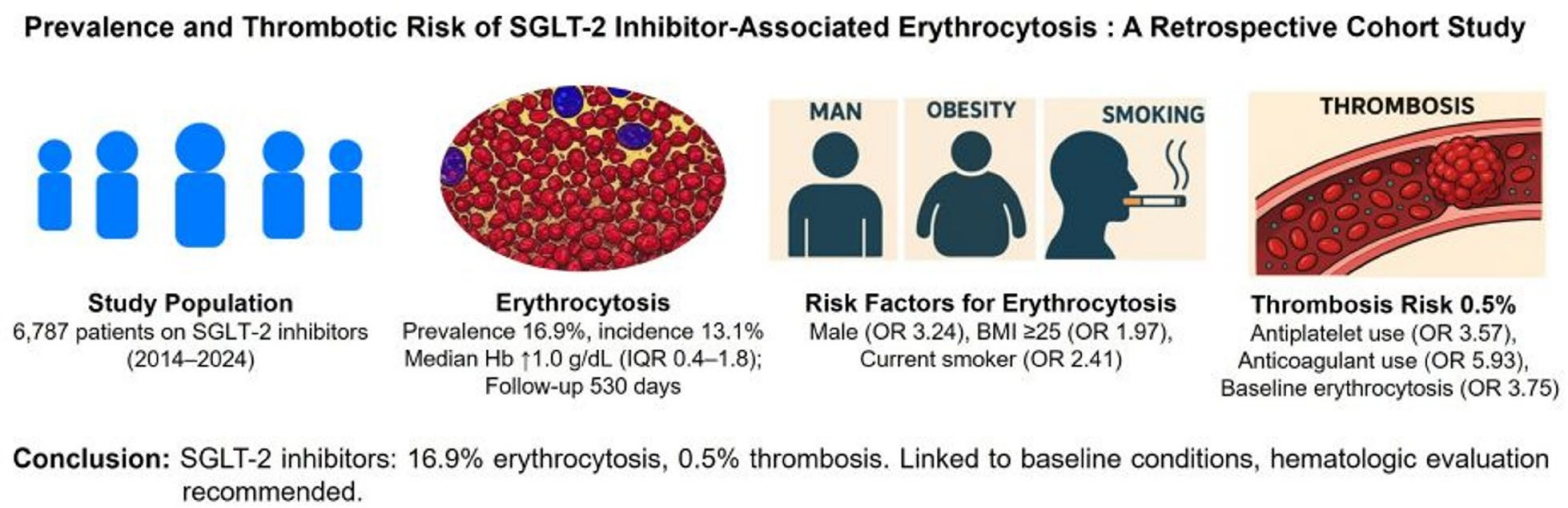

**Supplementary Information:**

The online version contains supplementary material available at 10.1186/s12933-025-02805-6.

## Research insights

**What is currently known about this topic?**

- SGLT-2 inhibitors manage diabetes, heart failure, and CKD.
- They may cause erythrocytosis, raising Hb levels.
- Thrombotic risk of erythrocytosis is unclear.

**What is the key research question?**

- What is the prevalence and thrombotic risk of SGLT-2 inhibitor-associated erythrocytosis?

**What is new?**

- 16.9% prevalence of erythrocytosis in SGLT-2 users.
- Male sex, obesity, smoking increase erythrocytosis risk.
- Thrombosis (0.5%) linked to baseline conditions.

**How might this study influence clinical practice?**

- Hematologic evaluation advised baseline erythrocytosis.

## Introduction

Sodium-glucose cotransporter 2 (SGLT-2) inhibitors have become a cornerstone therapy for managing type 2 diabetes mellitus (DM), heart failure (HF), and chronic kidney disease (CKD) due to their proven efficacy in glycemic control and decreasing cardiovascular events and heart failure hospitalizations as well as preventing the progression of kidney disease [1–3]. Accordingly, the American Diabetes Association updated its guidelines in 2022 to recommend SGLT-2 inhibitors as first-line treatment options for patients with heart and kidney disease [4]. Prescriptions of SGLT-2 inhibitors have significantly increased across various countries in recent years, with this trend being particularly pronounced among patients at risk for cardiovascular and renal diseases [5, 6]. Additionally, in Korea, SGLT-2 inhibitor prescriptions rose from 2.5% in 2015 to 13.9% in 2021 [7].

Recent clinical observations and case reports have increasingly associated SGLT-2 inhibitor use with erythrocytosis, characterized by elevated hemoglobin (Hb) and hematocrit (Hct) levels, leading to a rise in hematology referrals for further evaluation [8–13]. SGLT-2 inhibitors demonstrated an approximate mean Hb increase of 1 g/dL compared to placebo in patients with CKD treated with empagliflozin [14]. Recent studies on JAK2-negative SGLT-2 inhibitor-associated erythrocytosis patients have reported a median hemoglobin increase of 2.3–2.5 g/dL [11, 13]. Thromboembolic events have been reported in 2.4%–10% of SGLT-2 inhibitor-related cases, but their causal link to drug-induced erythrocytosis remains unconfirmed, and population-specific baseline risks are poorly characterized [15]. Doi et al. reported that canagliflozin may pose a thromboembolic risk in males with baseline erythrocytosis, highlighting the need for further investigation [16]. Despite this emerging link, the prevalence of SGLT-2 inhibitor-associated erythrocytosis remains poorly defined. Moreover, treatment patterns and the prognostic implications, including the risk of thrombosis, are inconsistent across studies, with conflicting evidence on whether elevated Hb contributes to thrombotic events. Therefore, this single-center retrospective study aims to determine the prevalence of erythrocytosis in patients receiving SGLT-2 inhibitors and to explore its association with thrombosis complications, providing critical insights into the clinical management of this population.

## Methods

### Patient selection

This single-center, retrospective cohort study was conducted at Seoul National University Bundang Hospital to investigate the prevalence and clinical outcomes of erythrocytosis associated with SGLT-2 inhibitors. Patients who received at least one of the following SGLT-2 inhibitors—dapagliflozin, empagliflozin, ertugliflozin, or ipragliflozin—between December 2014 and September 2024 were identified from electronic medical records (EMR).

Inclusion criteria comprised adults prescribed an SGLT-2 inhibitor for at least 3 consecutive months and with at least one Hb measurement following SGLT-2 inhibitor initiation, initially identifying 10,755 patients. Key exclusion criteria included no baseline Hb measurement within 2 months prior to starting the SGLT-2 inhibitor (n = 3661). A prescription was defined as an EMR entry for an SGLT-2 inhibitor with a planned duration of ≥ 3 months. Confirmed use was verified through follow-up clinic notes, prescription refills, or patient-reported adherence, ensuring continuous SGLT-2 inhibitor use for ≥ 3 months. Patients with documented use of < 3 months (n = 292) were excluded to ensure adequate exposure for assessing erythrocytosis risk. We excluded myeloproliferative neoplasms (MPNs), including polycythemia vera (PV), essential thrombocythemia, and myelofibrosis, identified via EMR review using World Health Organization (WHO) criteria. A patient with a confirmed PV diagnosis was excluded. Additional exclusion criteria are detailed in Supplementary Fig. 1. After applying these criteria, 6787 patients were included in the final cohort.

The study was carried out in accordance with the Declaration of Helsinki and was approved by the Institutional Review Board (IRB) of Seoul National University Bundang Hospital (IRB No. B-2404-892-402). The need for patient consent was waived by the IRB due to the retrospective nature of the study.

### Assessment and definition

Demographic and clinical data were extracted from EMR, including baseline characteristics such as age, sex, comorbidities, body mass index (BMI), smoking history, alcohol consumption history, and relevant medication history, as well as SGLT-2 inhibitor details such as specific agent prescribed, date of initiation, and actual duration of therapy. Laboratory data encompassed baseline Hb levels, defined as any measurement within 2 months prior to SGLT-2 inhibitor initiation, and follow-up Hb levels and other complete blood count (CBC) indices collected from peak time point after SGLT-2 inhibitor initiation. The purpose of SGLT-2 inhibitor prescriptions (DM, CKD, or HF) was determined from clinician-documented indications in prescription or clinic notes. Hematology consultations were identified via EMR referrals to the hematology department or documentation of hematologic evaluations (e.g., JAK2 mutation testing, bone marrow examination). Reasons for SGLT-2 inhibitor discontinuation were extracted from clinic notes, with “poor glycemic control” defined as inadequate glycemic control based on clinical judgment and “side effects” encompassing adverse events excluding erythrocytosis (e.g., urinary tract infections, renal function decline, dehydration, weight loss). The index date was defined as the initiation of SGLT-2 inhibitor therapy, identified from the EMR prescription entry. Follow-up began on the index date and continued until erythrocytosis, thrombosis, SGLT-2 inhibitor discontinuation, loss to follow-up, or the study end date (September 30, 2024), whichever occurred first.

Erythrocytosis was defined in accordance with standard diagnostic criteria as Hb > 16.5 g/dL or Hct > 49% in men, and Hb > 16.0 g/dL or Hct > 48% in women; for this study, any Hb exceeding these thresholds during follow-up was considered an event. Thrombotic events included arterial (ischemic stroke, transient ischemic attack, acute myocardial infarction, and peripheral arterial occlusion) and venous (lower extremity deep vein thrombosis, pulmonary embolism, hepatic vein thrombosis, portal vein thrombosis, mesenteric vein thrombosis, and cerebral vein thrombosis) events during SGLT-2 inhibitor treatment, confirmed by imaging (e.g., computed tomography, doppler ultrasound) or clinical documentation. The primary outcome was to evaluate the prevalence of erythrocytosis among patients receiving SGLT-2 inhibitors, with secondary outcomes including the analysis of risk factors associated with erythrocytosis in patients taking SGLT-2 inhibitors and the assessment of the prevalence and risk factors of thrombosis in patients with erythrocytosis induced by SGLT-2 inhibitors.

### Statistical analysis

Categorical variables were reported as frequencies (percentages), and continuous variables as means ± standard deviations or medians (interquartile ranges, IQR), based on data distribution. Baseline characteristics were compared between patients with and without erythrocytosis, and between those with and without thrombosis, using the Chi-square test or Fisher’s exact test for categorical variables, depending on expected cell counts. Continuous variables were assessed with appropriate parametric or nonparametric tests based on normality. Univariate analyses were performed to identify factors associated with erythrocytosis and thrombosis. Multivariable logistic regression models were used to identify independent risk factors for erythrocytosis or thrombosis, calculating odds ratios (ORs) with 95% confidence intervals (CIs) for each variable. Model fit was verified using the Likelihood Ratio Test, which was statistically significant (p < 0.001). Multicollinearity was assessed with the Variance Inflation Factor (VIF), with all variables showing VIF values < 5, indicating no multicollinearity concerns. All tests were two-sided, with statistical significance set at p < 0.05. Analyses were conducted using SPSS software (version 25.0, IBM Corp., USA).

## Results

### Trends in SGLT-2 inhibitor prescriptions by purpose

Supplementary Fig. 2 illustrates the trend of the average monthly prescription volume by year, categorized according to the purpose of SGLT-2 inhibitor prescriptions. Of the 6787 patients analyzed, 5805 (85.5%) were prescribed SGLT-2 inhibitors for DM, 550 (8.1%) for CKD, and 432 (6.4%) for HF. Prescriptions have been rapidly increasing since 2018, with the number of prescriptions in 2023 being approximately 7.4 times higher than in 2018. Additionally, both CKD and HF prescriptions exhibited a marked rise from 2020 onward.

### Baseline characteristics and prevalence of erythrocytosis

Among the 6787 patients included in this retrospective study at Bundang Seoul National University Hospital, 1145 (16.9%) developed erythrocytosis after initiating SGLT-2 inhibitor therapy, while 5642 (83.1%) did not. The median follow-up was 530 days (IQR, 277–981 days) for the overall cohort and 773 days (IQR, 445–1305 days) for the erythrocytosis subgroup. Table 1 summarizes the baseline characteristics of the study population, stratified by the presence of erythrocytosis. Patients with erythrocytosis were significantly younger, with only 11.2% being ≥ 70 years of age compared to 37.5% in the non-erythrocytosis group (p < 0.001). Males were more prevalent in the erythrocytosis group (88.9% vs. 60.0%, p < 0.001). A higher proportion of patients with erythrocytosis had a BMI ≥ 25 kg/m^2^ (63.9% vs. 48.1%, p < 0.001). Regarding comorbidities, DM was more frequent in patients with erythrocytosis (89.9% vs. 85.4%; p < 0.001). In contrast, comorbidities including hypertension (HTN), dyslipidemia (DL), HF, CKD, coronary artery disease (CAD), cerebrovascular accident (CVD), peripheral artery disease (PAD), and chronic obstructive pulmonary disease (COPD), were more prevalent in the non-erythrocytosis group (all p < 0.001).

**Table 1:** Baseline characteristics at SGLT-2 inhibitor initiation by erythrocytosis status during treatment

Variables	Total (n = 6787)	Without erythrocytosis (n = 5642)	With erythrocytosis (n = 1145)	p-value
Age ≥ 70 years; n (%)	2246 (33.1)	2118 (37.5)	128 (11.2)	< 0.001
Males; n (%)	4402 (64.9)	3384 (60.0)	1018 (88.9)	< 0.001
BMI ≥ 25 kg/m^2^; n (%)	3399 (50.1)	2625 (46.5)	774 (67.6)	< 0.001
*Comorbidity*; n (%)				
DM	5847 (86.1)	4818 (85.4)	1029 (89.9)	< 0.001
HTN	4447 (65.5)	3855 (68.3)	592 (51.7)	< 0.001
DL	3804 (56.0)	3,233 (57.3)	571 (49.9)	< 0.001
HF	1330 (19.6)	1181 (20.9)	149 (13.0)	< 0.001
CKD	2090 (30.8)	1,907 (33.8)	183 (16.0)	< 0.001
Coronary disease	2130 (31.4)	1838 (32.6)	292 (25.2)	< 0.001
CVA	1094 (16.1)	955 (16.9)	139 (12.1)	< 0.001
PAD	121 (1.8)	118 (2.1)	3 (0.3)	< 0.001
COPD	196 (2.9)	186 (3.3)	10 (0.9)	< 0.001
Sleep apnea	58 (0.9)	47 (0.8)	11 (1.0)	0.801
Current smoker; n (%)	1343 (19.8)	885 (15.7)	458 (40.0)	< 0.001
Current alcohol consumption; n (%)	1501 (22.1)	1098 (19.5)	403 (35.2%)	< 0.001
*Type of SGLT-2 inhibitor*; n (%)				0.590
Dapagliflozin	3884 (57.2)	3249 (57.6)	635 (55.5)	
Empagliflozin	2858 (42.1)	2355 (41.7)	503 (43.9)	
Ertugliflozin	31 (0.5)	26 (0.5)	5 (0.4)	
Ipragliflozin	14 (0.2)	12 (0.2)	2 (0.2)	
Antiplatelet agent use (yes); n (%)	2780 (41.0)	2364 (41.9)	416 (36.3)	0.001
Anticoagulation use (yes); n (%)	783 (11.5)	664 (11.4)	139 (12.1)	0.516
Baseline erythrocytosis (yes); n (%)	372 (5.5)	67 (1.2)	305 (26.6)	< 0.001

BMI, Body Mass Index; DM, Diabetes Mellitus; HTN, Hypertension; DL, Dyslipidemia; HF, Heart Failure; CKD, Chronic Kidney Disease; CVA, Cerebrovascular Accident; PAD, Peripheral Artery Disease; COPD, Chronic Obstructive Pulmonary Disease; SGLT-2, Sodium-Glucose Cotransporter 2

Age ≥ 70 years was based on EMR-recorded age at SGLT-2 inhibitor initiation. BMI ≥ 25 kg/m^2^ was calculated from height and weight at baseline (WHO obesity threshold for Asian populations). Comorbidities (DM, HTN, DL, HF, CKD, CAD, CVA, PAD, COPD, sleep apnea) were identified using clinician diagnoses in EMR. Current smoking and alcohol consumption were based on patient-reported history within the past 6 months. Antiplatelet and anticoagulant use was defined as active prescriptions at baseline. Baseline erythrocytosis was defined as Hb > 16.5 g/dL or Hct > 49% (men), or Hb > 16.0 g/dL or Hct > 48% (women), within 2 months prior to SGLT-2 inhibitor initiation

Current smoking (40.0% vs. 15.7%) and alcohol consumption (35.2% vs. 19.5%) were more prevalent in the erythrocytosis group (both p < 0.001). SGLT-2 inhibitor type did not differ (p = 0.590), with dapagliflozin most common (57.2%). Antiplatelet use was less frequent in the erythrocytosis group (36.3% vs. 41.9%; p = 0.001), while anticoagulation was similar (12.1% vs. 11.4%, p = 0.516). Baseline erythrocytosis was significantly more common in the erythrocytosis group than in the non-erythrocytosis group (26.6% vs. 1.2%; p < 0.001). In the analysis excluding patients with baseline erythrocytosis (n = 372), the incidence of erythrocytosis was 13.1% (n = 840/6415).

### Multivariable analysis of risk factors for erythrocytosis

To address the potential impact of baseline erythrocytosis, a sensitivity analysis was performed by excluding patients with baseline erythrocytosis (n = 372). The remaining cohort (n = 6415) was analyzed for erythrocytosis risk factors. Multivariable analysis identified several risk factors for erythrocytosis (Table 2). Male sex (OR 3.24, 95% CI 2.47–4.26), BMI ≥ 25 kg/m^2^ (OR 1.97, 95% CI 1.63–2.39), and current smoking (OR 2.41, 95% CI 1.96–2.96) were strongly associated with increased risk (all p < 0.001). Conversely, age ≥ 70 years (OR 0.46, 95% CI 0.35–0.59), HTN (OR 0.71, 95% CI 0.58–0.86), DL (OR 0.67, 95% CI 0.55–0.81), and CKD (OR 0.51, 95% CI 0.40–0.66) were associated with reduced risk. PAD, and COPD also showed protective effects (Table 2).

**Table 2:** Multivariable analysis of risk factors for erythrocytosis during SGLT-2 inhibitors

	Odd ratio	95% CI	p-value
Age ≥ 70 years	0.46	0.35–0.59	< 0.001
Male sex	3.24	2.47–4.26	< 0.001
BMI ≥ 25 kg/m^2^	1.97	1.63–2.39	< 0.001
Current smoker	2.41	1.96–2.96	< 0.001
HTN	0.71	0.58–0.86	0.001
DL	0.67	0.55–0.81	< 0.001
CKD	0.51	0.40–0.66	< 0.001
PAD	0.11	0.02–0.79	0.028
COPD	0.38	0.16–0.89	0.025

BMI, Body Mass Index; HTN, Hypertension; DL, Dyslipidemia; CKD, Chronic Kidney Disease; PAD, Peripheral Artery Disease; COPD, Chronic Obstructive Pulmonary Disease

### Effect of SGLT-2 inhibitors on hematologic parameters

**Fig. 1:**
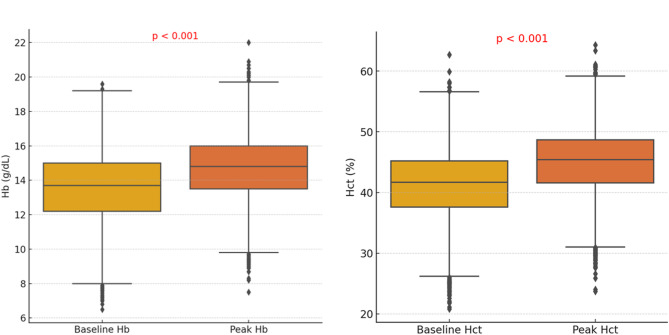
Changes in hematologic parameters from baseline to peak levels

**Fig. 2:**
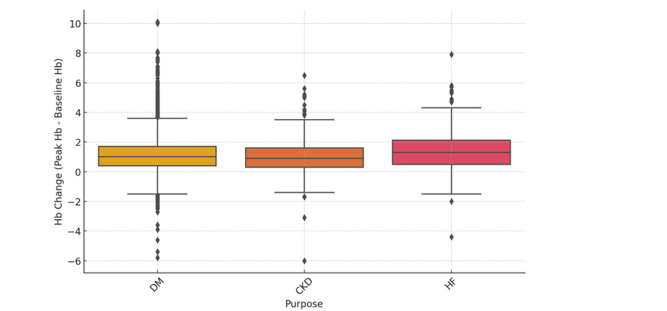
Hemoglobin change across different purposes

**Fig. 3:**
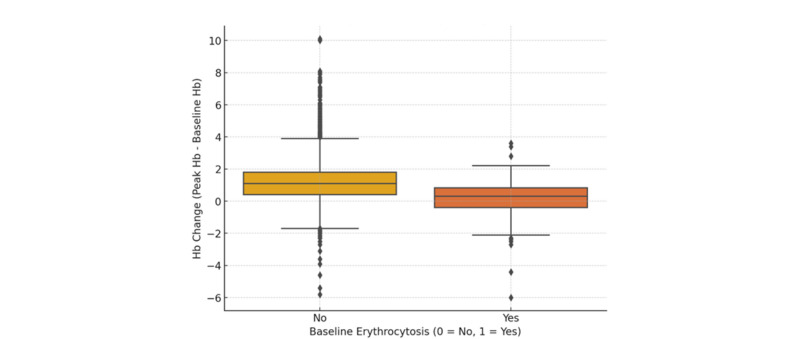
Hemoglobin change by baseline erythrocytosis status

Figure 1 illustrates the changes in Hb and Hct from baseline to peak levels in the total cohort. The median Hb increase for the entire cohort was 1.0 g/dL (IQR, 0.4–1.8 g/dL, p < 0.001), and the median Hct increase was 3.5% (IQR, 1.4–5.7%, p < 0.001). The median time to peak Hb was 210 days (IQR, 109–434 days) following SGLT-2 inhibitor initiation. Compared to baseline Hb and Hct, peak Hb and Hct showed a statistically significant increase, indicating a significant hematologic response to SGLT-2 inhibitor therapy, consistent with the known erythropoietic effects of these agents. Figure 2 shows that the change in Hb levels significantly differs based on the purpose of treatment (p < 0.05). CKD patients showed a median Hb increase of 0.9 (IQR, 0.3–1.6), while DM patients had a median increase of 1.0 (IQR, 0.4–1.7). HF patients exhibited the highest median Hb increase at 1.3 (IQR, 0.5–2.1). This indicates that HF patients experienced the most significant rise in Hb levels, whereas CKD patients showed the lowest increase. Patients without baseline erythrocytosis exhibited significantly greater Hb changes compared to those with erythrocytosis (p < 0.05). The median Hb change was 1.1 (IQR, 0.4–1.8) in patients without erythrocytosis, whereas it was 0.3 (IQR, -0.4–0.8) in those with erythrocytosis (Fig. 3). Table 3 summarizes the hematologic parameters at baseline and peak levels for the total cohort (n = 6787), stratified by sex. Significant sex differences were observed in both baseline and peak Hb and Hct levels, with males consistently exhibiting higher values than females.

**Table 3:** Hematologic parameters at baseline and peak levels by sex during SGLT-2 inhibitor use

Category	Total (median, rage)	Male (median, rage)	Female (median, rage)
Baseline Hb (g/dL)	13.7 (12.2–15.0)	14.3 (12.8–15.4)	12.8 (11.6–13.9)
Baseline Hct (%)	41.7 (37.6–45.2)	43.2 (39.0–46.4)	39.4 (36.0–42.3)
Peak Hb (g/dL)	14.8 (13.5–16.0)	15.4 (14.1–16.5)	14.0 (12.8–14.9)
Peak Hct (%)	45.4 (41.6–48.7)	46.8 (43.0–49.8)	43.2 (39.9–45.8)

Hb, Hemoglobin; Hct, Hematocit

**Fig. 4:**
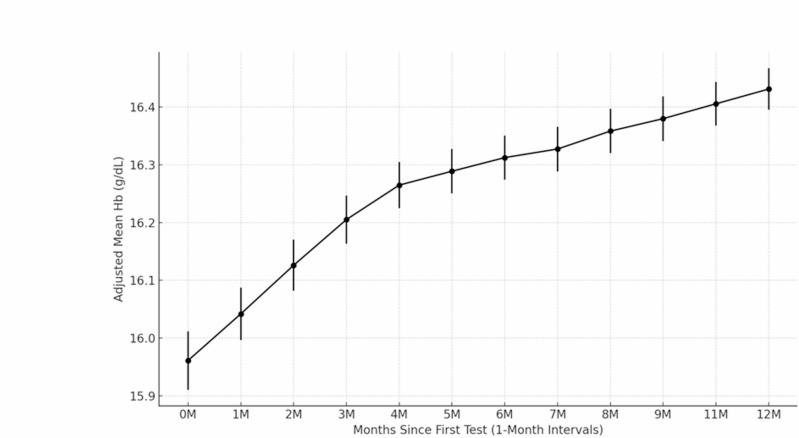
12-month hemoglobin trends

Among patients with erythrocytosis (n = 1145), the median hemoglobin increase was 1.4 g/dL (IQR, 0.8–2.2), and the median hematocrit increase was 4.7% (IQR, 2.7–6.8). The median time to peak hemoglobin was 361 days (IQR, 161–634). Serial Hb changes were assessed over 12 months in 769 patients receiving SGLT-2 inhibitors with at least 12 months of Hb follow-up data (Fig. 4). Hb levels rose rapidly within the first 3 months, followed by a more gradual increase, maintaining an overall upward trend over the 12-month period.

Of the 6787 patients, 4756 (70.1%) continued treatment, 944 (13.9%) were lost to follow-up or referred out, and 1087 (16.0%) discontinued treatment. Among the 983 patients with known reasons for discontinuation (excluding 104 patients with unknown reasons), the most common were poor DM control (34.7%), side effects other than erythrocytosis (32.2%), and diabetes medication reduction (14.3%), with erythrocytosis accounting for only 5 (0.5%) discontinuations.

Among patients who developed erythrocytosis while using SGLT-2 inhibitors, hematology consultations occurred in 18 (1.6%) patients, with median serum erythropoietin (EPO) levels of 12.5 mIU/mL (range 7.1–17.61 mIU/mL, reference 2.59–18.50 mIU/mL). Of these, 10 (55.6%) underwent JAK2 mutation testing, 4 (22.2%) had bone marrow examinations, 2 (11.1%) received phlebotomy, and 2 (11.1%) were prescribed aspirin. Four patients discontinued SGLT-2 inhibitors: two due to erythrocytosis (one resolved spontaneously, and one improved with phlebotomy) and two due to well-controlled diabetes (Supplementary Fig. 3).

### Thrombosis risk factors

Among the 6787 patients, 0.5% (33 patients) developed thrombosis during the treatment period with SGLT-2 inhibitors (Table 4). HF was more common in the thrombosis group (33.3% vs. 19.5%, p = 0.046), as were antiplatelet use (63.6% vs. 40.8%, p = 0.008), anticoagulant use (36.4% vs. 11.4%, p < 0.001), and baseline erythrocytosis (15.2% vs. 5.4%, p = 0.032). Other factors, including age ≥ 70 years, sex, BMI, eGFR, most comorbidities (e.g., DM, HTN. CAD), smoking, and alcohol use, showed no significant differences between groups.

**Table 4:** Baseline characteristics at the time of SGLT-2 inhibitor initiation in patients with versus without thrombosis during treatment

Variables	Total (n = 6,787)	Without thrombosis (n = 6,754)	With thrombosis (n = 33)	p-value
Age ≥ 70 (%)	2246 (33.1%)	2234 (33.1%)	12 (36.4%)	0.689
Males; n (%)	4402 (64.9%)	4378 (64.8%)	24 (72.7%)	0.343
BMI ≥ 25, kg/m^2^; n (%)	3399 (50.9%)	3381 (50.9%)	18 (54.5%)	0.672
*Comorbidity*; n (%)				
DM	5847 (86.1%)	5817 (86.1%)	30 (90.9%)	0.428
HTN	4447 (65.5%)	4,429 (65.6%)	18 (54.5%)	0.184
DL	3804 (56.0%)	3,788 (56.1%)	16 (48.5%)	0.380
HF	1330 (19.6%)	1319 (19.5%)	11 (33.3%)	0.046
CKD	2090 (30.8%)	2081 (30.8%)	9 (27.3%)	0.660
Coronary disease	2130 (31.4%)	2115 (31.3%)	15 (45.5%)	0.081
CVA	1094 (16.1%)	1087 (16.1%)	7 (21.2%)	0.425
PAD	121 (1.8%)	121 (1.8%)	0 (0.0%)	1.000
COPD	196 (2.9%)	196 (2.9%)	0 (0.0%)	1.000
Sleep apnea	58 (0.9%)	58 (0.9%)	0 (0.0%)	1.000
Current smoker	1343(19.8%)	1331 (19.7%)	12 (36.4%)	0.127
Current alcohol consumption	1501(22.1)	1493 (22.1%)	8 (24.4%)	0.584
Antiplatelet agent use	2780 (41.0%)	2759 (40.8%)	21 (63.6%)	0.008
Anticoagulant use	783 (11.5%)	771 (11.4%)	12 (36.4%)	< 0.001
Baseline erythrocytosis	372 (5.5)	367 (5.4)	5 (15.2)	0.032

BMI, Body Mass Index; DM, Diabetes Mellitus; HTN, Hypertension; DL, Dyslipidemia; HF, Heart Failure; CKD, Chronic Kidney Disease; CVA, Cerebrovascular Accident; PAD, Peripheral Artery Disease; COPD, Chronic Obstructive Pulmonary Disease; SGLT-2, Sodium-Glucose Cotransporter 2

Antiplatelet agent use (OR 3.57, 95% CI 1.60–7.97, p = 0.002), anticoagulant use (OR 5.93, 95% CI 2.60–13.57, p < 0.001), and baseline erythrocytosis (OR 3.75, 95% CI 1.41–9.96, p = 0.008) were significantly linked to an increased risk of thrombosis in multivariable analysis (Table 5).

**Table 5:** Multivariable analysis of risk factors for thrombosis

	Odd ratio	95% CI	p-value
Antiplatelet agent use	3.57	1.60–7.97	0.002
Anticoagulant use	5.93	2.60–13.57	< 0.001
Baseline erythrocytosis	3.75	1.41–9.96	0.008

Among 33 patients, five patients had erythrocytosis at the time of the thrombosis event and had experienced at least one episode of erythrocytosis within the preceding six months. Notably, all five patients exhibited arterial thrombosis without any venous thrombosis events. However, these five patients also had underlying conditions (atrial fibrillation, severe coronary calcification, large artery atherosclerosis) associated with their thrombosis events, making it unlikely that the thrombosis events were directly related to erythrocytosis. Thrombotic events occurred in 1.8% (n = 21) of the erythrocytosis group, compared with 0.2% (n = 12) in the non-erythrocytosis group (p < 0.001). Within the erythrocytosis group, peak Hb levels were stratified into tertiles: low (16.1–16.9 g/dL), medium (16.9–17.5 g/dL), and high (17.5–22.0 g/dL). Thrombosis rates were 1.4% (n = 6), 2.2% (n = 8), and 2.0% (n = 7), respectively (p = 0.649), suggesting no clear dose–response relationship between Hb levels and thrombosis.

## Discussion

This retrospective study examined the prevalence of erythrocytosis and its association with thrombosis in 6787 patients treated with SGLT-2 inhibitors over a median follow-up of 530 days. We observed that 16.9% of patients developed erythrocytosis, with a median Hb increase of 1.0 g/dL and Hct increase of 3.5% in our cohort, consistent with recent reports linking SGLT-2 inhibitors to elevated Hb and Hct levels [2, 17]. The Hb increase observed in our study was lower than the median increase of 2.3–2.5 g/dL reported by Gangat et al.[11] and Liu et al.[13], a discrepancy that may stem from their focus on patients with confirmed SGLT-2 inhibitor-associated erythrocytosis, potentially enriching their cohort with individuals showing more pronounced responses. The variation in Hb increases by treatment purpose—highest in HF patients (1.3 g/dL), followed by DM (1.0 g/dL) and CKD (0.9 g/dL)—suggests that underlying disease states modulate the hematologic response. The greater Hb rise in HF patients may be linked to improved oxygenation due to reduced fluid overload, while the lower increase in CKD patients could reflect baseline anemia or impaired erythropoietin production [18, 19]. Patients without baseline erythrocytosis exhibited significantly greater Hb changes compared to those with erythrocytosis (p < 0.05), a finding that aligns with a Danish population study reporting a more pronounced Hb increase in patients without baseline erythrocytosis and suggests enhanced erythropoietic responsiveness [20].

Multivariable analysis identified younger age, male sex, higher BMI, and current smoking as significant risk factors for erythrocytosis, consistent with recent findings by Nguyen et al.[21]. However, a significant limitation is that only 1.6% of erythrocytosis patients underwent hematology consultations, meaning that comprehensive diagnostic evaluations—including assessments of EPO levels, androgen status, and JAK2 mutation status—were not performed in most patients with baseline erythrocytosis, potentially allowing underlying hematologic conditions like PV to be included in this group; indeed, Koseoglu et al. reported that 13.3% of erythrocytosis patients were JAK2 mutation-positive [22]. Older age, HTN, DL, CKD, and PAD may contribute to anemia of chronic disease, potentially offsetting SGLT-2 inhibitor effects and reducing erythrocytosis risk [23]. The lower erythrocytosis risk in COPD may result from anemia of chronic disease due to persistent inflammation. Although secondary erythrocytosis is a known compensatory response to hypoxemia in COPD, recent studies indicate that systemic inflammation may reduce hemoglobin levels in these patients [24–26].

There is no definitive evidence that secondary polycythemia increases the risk of thrombosis [27]. In a large population-based series, erythrocytosis—defined by the higher 2008 WHO criteria for Hgb and Hct thresholds—was associated with increased cardiovascular morbidity and mortality; however, this association was not observed when broader criteria were applied [28]. As a cross-sectional study, it cannot establish causality. Stratification by Hb levels within the erythrocytosis group in this study revealed no significant association with thrombosis risk, consistent with Gangat et al.'s finding of no direct link between Hb levels and thrombosis in SGLT-2 inhibitor-associated erythrocytosis [11]. Regarding thrombosis risk, the low incidence (0.5%) suggests that SGLT-2 inhibitor-induced erythrocytosis does not substantially elevate thrombotic events in this cohort. Our multivariable analysis identified antiplatelet use, anticoagulant use, and baseline erythrocytosis as significant risk factors for thrombosis in patients on SGLT-2 inhibitors, likely reflecting underlying thrombotic predispositions. The association between antiplatelet or anticoagulant use and increased thrombotic risk suggests confounding by indication, as patients prescribed these medications likely have a higher baseline thrombotic risk. Anticoagulant use was identified from EMR prescription records, primarily for stroke and embolism prevention in non-valvular atrial fibrillation or as combination therapy with aspirin in CAD or PAD. This use was linked to increased thrombosis risk, probably reflecting underlying thrombotic predispositions in these patients. However, the lack of baseline thrombosis history assessment limits our ability to discern causality, and the association with baseline erythrocytosis is confounded by insufficient investigation into its causes—such as EPO levels or JAK2 mutation status—as only 1.6% of erythrocytosis patients received hematology consultations, potentially missing underlying conditions like PV. Consequently, patients presenting with baseline erythrocytosis warrant a comprehensive medical evaluation and hematologic consultation prior to initiating SGLT-2 inhibitor. Although thrombotic events were statistically more frequent in the erythrocytosis group, clinical review showed that only five patients had erythrocytosis at the time of the event. Most of these patients had established prothrombotic comorbidities, including atrial fibrillation, severe coronary artery calcification, or large artery atherosclerosis. This suggests that the observed association may be partly driven by confounding factors rather than a direct causal link. These findings highlight the need for thorough evaluation of at-risk patients.

This study has limitations. Its retrospective, single-center design may limit generalizability, as erythrocytosis and thrombosis rates could differ across diverse populations or healthcare settings. We acknowledged the limitation of the retrospective design, as systematic exclusion of MPNs relied on EMR-documented diagnoses, and only 10 of 1145 erythrocytosis patients underwent JAK2 mutation testing, potentially missing MPN cases. Testosterone therapy, a known risk factor for erythrocytosis, was not evaluated due to incomplete EMR data on hormone replacement therapies. Among the 18 erythrocytosis patients who received hematology consultations, no testosterone-related erythrocytosis cases were identified. However, the lack of comprehensive testosterone use assessment across the entire cohort remains a limitation. EPO use, a known stimulator of erythropoiesis, was also not assessed due to incomplete EMR data on its administration, representing a potential confounder, particularly in patients with comorbidities such as CKD. Uncollected information on erythrocytosis risk factors—such as familial history, history of renal transplantation, endocrine disorders, uterine myoma, and EPO-producing tumors—may have influenced the study results. Defining the erythrocytosis group as patients with at least one episode of erythrocytosis may be overly broad, potentially complicating result interpretation. Only 22.8% of patients underwent hemoglobin Hb monitoring for over 36 months during SGLT-2 inhibitor therapy. Future studies should use serial CBC data to assess the frequency and duration of erythrocytosis episodes to better evaluate its clinical significance. Thrombosis was rare (0.5%) and primarily linked to baseline conditions; however, the retrospective design and limited number of events (n = 33) prevent definitively excluding a contribution from SGLT-2 inhibitor-associated erythrocytosis. Additionally, in this retrospective study, CBC data were not collected at regular intervals, making it challenging to accurately track the erythrocytosis status prior to thrombotic events and thus limiting our ability to assess the temporal relationship between erythrocytosis and thrombosis.

In this Korean cohort, SGLT-2 inhibitors were associated with a 16.9% prevalence of erythrocytosis and a 13.1% incidence in patients without baseline erythrocytosis, with male sex, obesity, and smoking as key risk factors. The low thrombosis rate (0.5%) indicates minimal thrombotic risk in this cohort. However, the retrospective design and small number of thrombotic events limit definitive safety conclusions. Targeted hematologic evaluation is recommended for patients with baseline erythrocytosis before initiating SGLT-2 inhibitors.

## Electronic supplementary material

Below is the link to the electronic supplementary material.

Supplementary Material 1

## Data Availability

The data that support the findings of this study are available on request from the corresponding author.
