# Supplementary material for: Prevalence and thrombotic risk of SGLT-2 inhibitor-associated erythrocytosis: a retrospective cohort study
Source: Cardiovasc Diabetol. 2025 Jul 10;24:276. doi: 10.1186/s12933-025-02805-6 (PMC12243421; doi:10.1186/s12933-025-02805-6)

**Prevalence and Thrombotic Risk of SGLT-2 Inhibitor-Associated Erythrocytosis: A Retrospective Cohort Study**

Ji Yun Lee^1^, Ju-Hyun Lee^1^, Eun-Jung Jung^1^, Woochan Park^1^, Jeongmin Seo^1^, Minsu Kang^1^, Eun Hee Jung^1^, Sang-A Kim^1^, Koung Jin Suh^1^, Ji-Won Kim^1^, Se Hyun Kim^1^, Jeong-Ok Lee^1^, Jin Won Kim^1^, Yu Jung Kim^1^, Keun-Wook Lee^1^, Jee Hyun Kim^1^, Soo-Mee Bang^1^†

^1^Department of Internal Medicine, Seoul National University College of Medicine, Seoul National University Bundang Hospital, Seongnam, Republic of Korea

Running title: SGLT-2 inhibitor and Erythrocytosis

**Address for correspondence:** Soo-Mee Bang, MD, PhD

Department of Internal Medicine, Seoul National University College of Medicine, Seoul National University Bundang Hospital, Gumi-ro 173 Beon-gil, Bundang-gu, Seongnam-Si, Gyeonggi-do 13620, Korea

Tel: +82-31-787-7039; Fax: +82-31-787-4098

E-mail: smbang7@snu.ac.kr

**Supplementary Figure 1. Patient Selection Process**


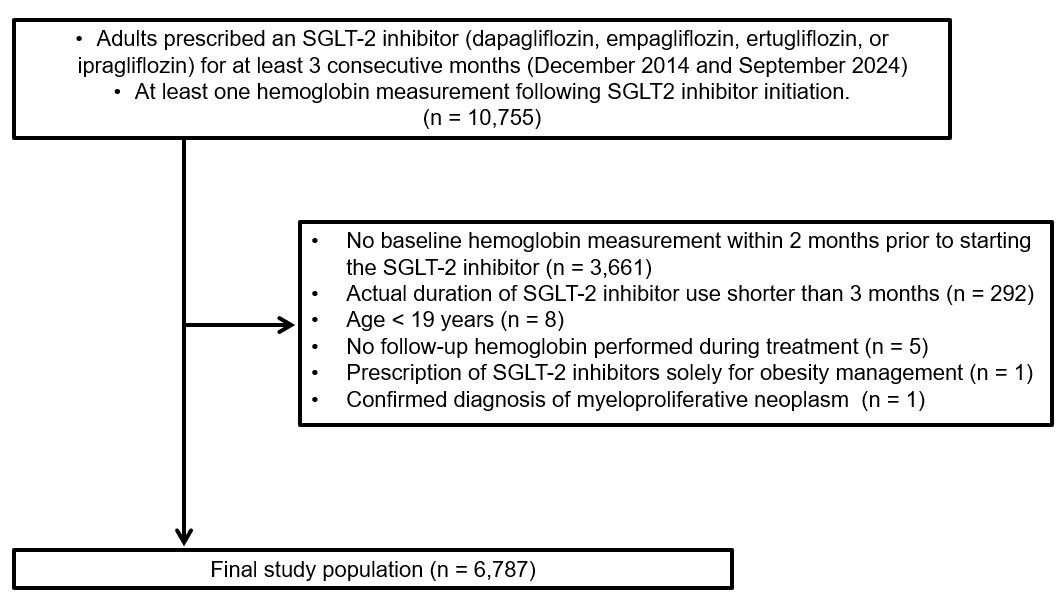


**Supplementary Figure 2. Average Monthly Prescription Volume by Year and Purpose**


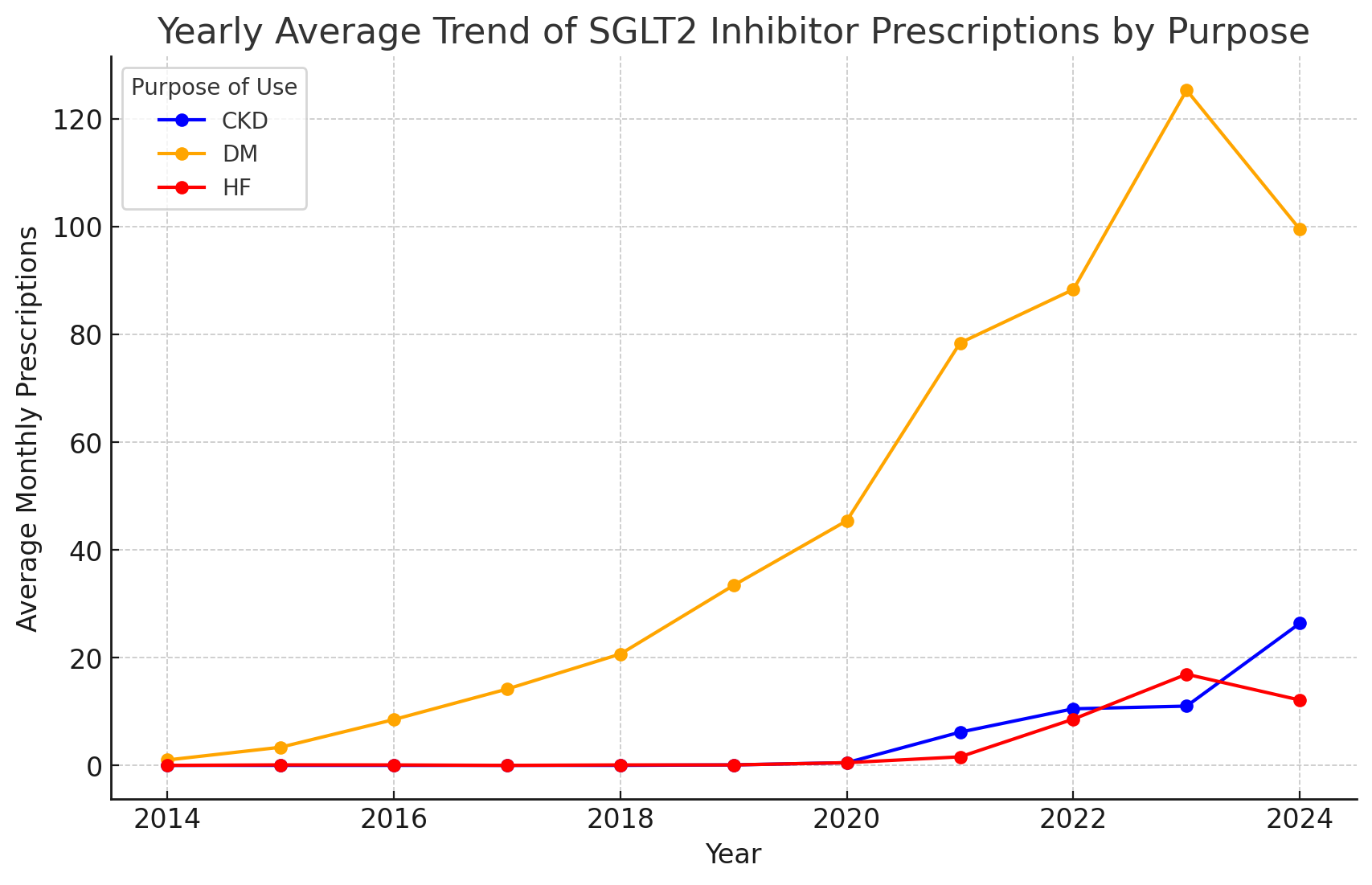


**Supplementary Figure 3. Patient Trajectory Following Erythrocytosis After SGLT-2 Inhibitor Use**


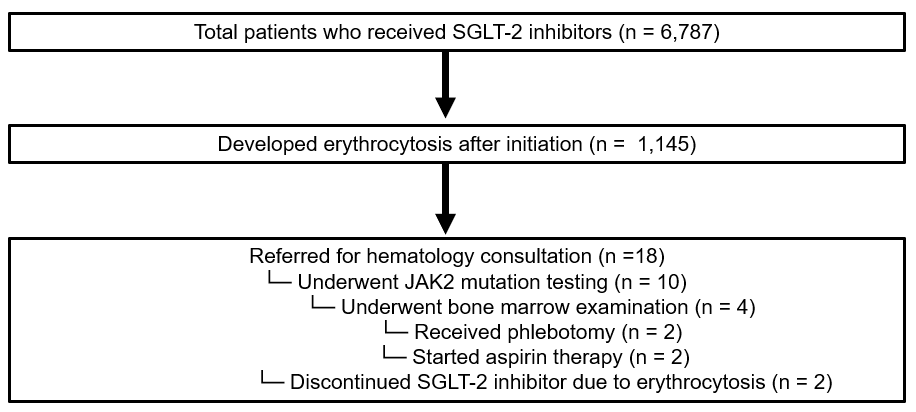

Supplement: Supplementary file 1 — Supplementary Material 1 [file 12933_2025_2805_MOESM1_ESM.docx]
